# Paclitaxel exposure downregulates miR-522 expression and its downregulation induces paclitaxel resistance in ovarian cancer cells

**DOI:** 10.1038/s41598-020-73785-8

**Published:** 2020-10-07

**Authors:** Mayuko Miyamoto, Kenjiro Sawada, Koji Nakamura, Akihiko Yoshimura, Kyoso Ishida, Masaki Kobayashi, Aasa Shimizu, Misa Yamamoto, Michiko Kodama, Kae Hashimoto, Tadashi Kimura

**Affiliations:** 1 Department of Obstetrics and Gynecology, Osaka University Graduate School of Medicine, 2-2, Yamadaoka, Suita, Osaka 5650871 Japan; 2 Department of Molecular Oncology, H. Lee Moffitt Cancer Center & Research Institute, 12902 Magnolia Dr, Tampa, FL 33612 USA

**Keywords:** Cancer, Cancer genetics, Gynaecological cancer

## Abstract

Paclitaxel resistance is a critical challenge in ovarian cancer treatment. This study aimed to identify microRNAs (miRNAs) that modulate paclitaxel resistance for use as potential therapeutic targets in such settings. Paclitaxel-resistant cell lines were established using two ovarian cancer cell lines: SKOV3ip1 and HeyA8. The evaluation of miRNA polymerase chain reaction (PCR) arrays indicated that the expression of miR-522-3p was downregulated in paclitaxel-resistant cells. The restoration of miR-522-3p sensitized the resistant cells to paclitaxel, and its downregulation desensitized the parental cells. Using PCR arrays, we focused on *E2F2,* with the luciferase reporter assay revealing that it was a direct target for miR-522-3p. The paclitaxel-resistant cells showed stronger *E2F2* expression than the parental cells, while *E2F2* inhibition sensitized the resistant cells to paclitaxel. Forced *E2F2* expression in the parental cells led to the acquisition of paclitaxel resistance, while miR-522-3p inhibited *E2F2* expression and was associated with retinoblastoma protein phosphorylation attenuation, which resulted in G0/G1 arrest. The effects of miR-522-3p and *E2F2* in ovarian cancer were examined using public databases, revealing that low miR-522-3p expression and high *E2F2* expression were associated with significantly poorer overall survival. In conclusion, miR-522-3p attenuated the degree of paclitaxel resistance in vitro through the downregulation of *E2F2;* miR-522-3p supplementation may be a therapeutic target for paclitaxel-resistant ovarian cancer.

## Introduction

Ovarian cancer is the most lethal gynecological malignancy and was the fifth leading cause of cancer-related death among American women in 2018^1^. Standard therapies for ovarian cancer include cytoreductive surgery and intensive adjuvant chemotherapy using a combination of taxane and platinum. Despite these multimodal treatments, patients with advanced-stage disease (approximately 70% of all cases) eventually experience recurrence, and the 5-year survival rate remains at 30%, without any dramatic improvements over the last 20 years^1^.

The taxane-platinum combination is used in first-line chemotherapy for ovarian cancer owing to its high efficacy and associated tolerable adverse effects^2^. Patients initially respond well to this regimen, although more than two-thirds of those with advanced-stage disease eventually experience relapse, often in relation to chemoresistance acquisition^3^. Therefore, paclitaxel plays an indispensable role in ovarian cancer treatment, and paclitaxel resistance often determines patients’ outcomes. The determination of the mechanisms that lead to paclitaxel chemoresistance is critical.

MicroRNAs (miRNAs) are small non-coding RNAs with a length of 20–25 nucleotides that regulate gene expression through the repression of the translation of their target genes or degradation of their target mRNAs by binding to the 3′-untranslated region (UTR) of the target genes^4^. More than 50% of miRNA target genes are located in cancer-associated genomic regions or in fragile sites, suggesting that miRNAs are deeply involved in cancer pathogenesis^5^. Furthermore, miRNAs are reportedly important modulators of chemoresistance^6^, through the regulation of apoptosis, expression of multiple drug resistance-related proteins, and the conversion to tumor stem-like cells in a variety of cancers^7^. A recent review identified a list of miRNAs (miR-27a, miR-182, miR-663, miR-31, and miR-106a) that may be related to paclitaxel resistance^8^.

Based on these concepts, we aimed to identify miRNAs that modulate paclitaxel resistance in vitro, and perform preliminary analyses of their potential as therapeutic targets.

## Results

### Establishment of paclitaxel-resistant ovarian cancer cell lines

**Figure 1:**
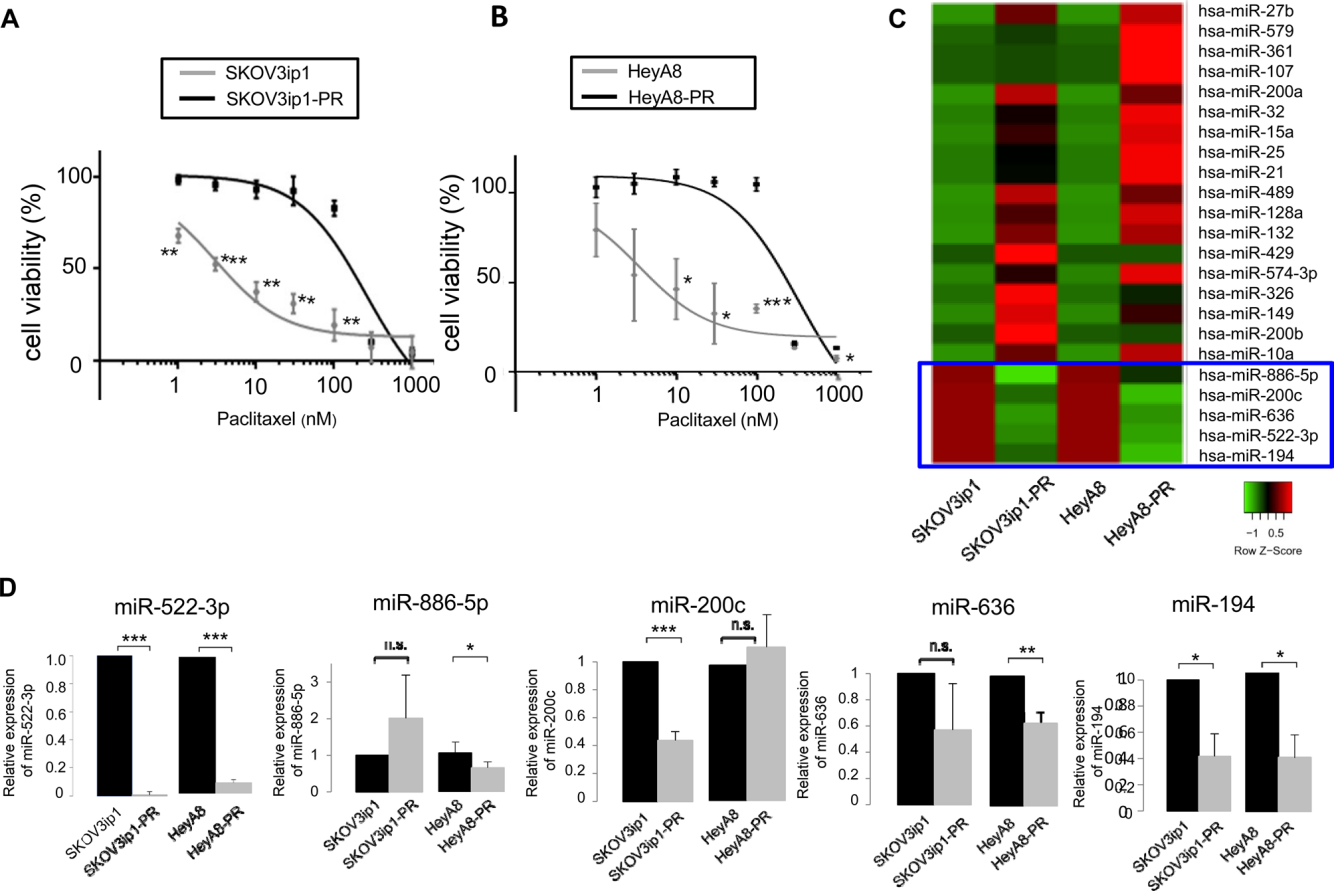
miR-522-3p expression is downregulated in paclitaxel-resistant ovarian cancer cells, and this lower expression is correlated with prognoses in ovarian cancer. **(A)** In vitro cell survival in response to paclitaxel treatment was determined using the MTS assay and SKOV3ip1/SKOV3ip1-PR cells (data are presented as mean ± standard deviation [SD], as obtained from three independent experiments). **(B)** Response of the HeyA8/HeyA8-PR cell to paclitaxel based on the MTS assay. Experiments were performed in triplicate. Data are represented as mean ± SD and were obtained from three independent experiments. **(C)** Heatmap of miRNA expression in both paclitaxel-resistant cell lines relative to their corresponding parental cell lines (red: > twofold, green: < 0.5-fold, blue squares: expression of miRNAs downregulated in both paclitaxel-resistant cell lines). **(D)** The miRNA quantitative RT-PCR findings, present as fold differences, with miR-522-3p, miR-886-5p, miR-200c, miR-636 and miR-194 expressed relative to RNU6B expression and calculated using the 2^−ΔΔCT^ method. n.s. not significant, *P < 0.05, **P < 0.01, and ***P < 0.001.

Using paclitaxel-resistant ovarian cancer cell lines—SKOV3ip1-PR and HeyA8-PR—we calculated the half maximal inhibitory concentration (IC_50_) values for paclitaxel. The paclitaxel-resistant cell lines had much higher IC_50_ values for paclitaxel (SKOV3ip1-PR: 259.2 nM, HeyA8-PR: 340.0 nM) than the parental SKOV3ip1 cells (3.0 nM) and parental HeyA8 cells (3.5 nM) (Fig. 1A,B).

### miR-522-3p expression is downregulated in paclitaxel-resistant ovarian cancer cells

Taqman miRNA arrays were created using SKOV3ip1, HeyA8, SKOV3ip1-PR, and HeyA8-PR cells, and the resulting data were assigned the GSE139043 identifier^9^. Relative to the parental cells, the paclitaxel-resistant cells had expression values that were < 0.5 for miR-522-3p, miR-194, miR-200c, miR-636, and miR-886-5p (Fig. 1C). Validation analyses based on miRNA reverse transcription polymerase chain reaction (RT-PCR) revealed that the expression of miR-522-3p and miR-194^10^ was significantly downregulated (Fig. 1D), while that of the others was not. Since the miR-522-3p expression level in the paclitaxel-resistant cells was drastically lower than that in the parental cells (SKOV3ip1-PR; 0.009-fold, HeyA8-PR; 0.08-fold), we focused on miR-522-3p in this study.

### Lower miR-522-3p expression is associated with response to paclitaxel plus platinum-based chemotherapy and poor prognoses

**Figure 2:**
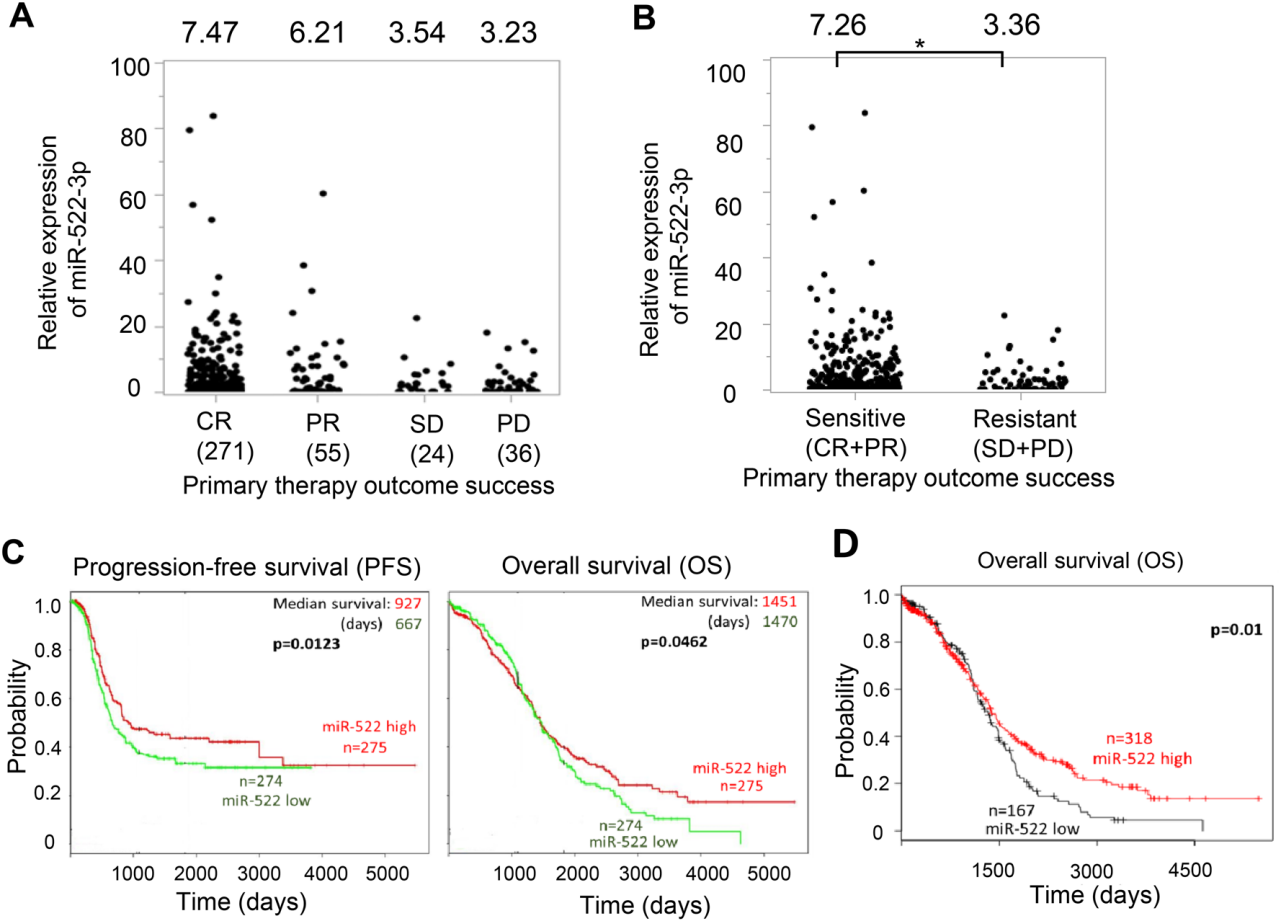
Lower miR-522-3p expression is associated with chemoresistance to paclitaxel plus platinum-based chemotherapy and poor prognoses in ovarian cancer. **(A)** miR-522-3p expression values in 489 high grade serous ovarian carcinoma patients deposited in the The Cancer Genome Atlas (TCGA) database. Based on the data, patients were classified as having complete response (CR), partial response (PR), stable disease (SD), or progressive disease (PD). **(B)** According to the miR-522-3p expression values in the TCGA database, we classified each patient as chemotherapy sensitive (CR + PR) or resistant (SD + PD). *P < 0.05. **(C)** Kaplan–Meier plot curves for progression-free survival (PFS; left) and overall survival (OS; right) among patients with serous ovarian cancer (n = 549). Patients were stratified according to the upper and lower tertile thresholds for the miR-522 probe in the PROGNOSTIC miRNA DATABASE. **(D)** The Kaplan–Meier curve for OS among 485 ovarian cancer patients.

First, to analyze the effect of miR-522-3p expression on the success rate of primary chemotherapy, data on patients’ clinical outcomes and the corresponding miR-522-3p expression levels were obtained from The Cancer Genome Atlas (TCGA) database, which includes 489 high-grade serous ovarian carcinoma patients. Of 489 patients, 271 achieved complete response (CR) and 55 showed partial response (PR) to primary chemotherapy, which predominantly comprised paclitaxel and carboplatin, while 24 showed stable disease (SD) and 36 progressive disease (PD). The average miR-522-2p values of those who achieved CR and PR were 7.47 and 6.21, respectively, although those of patients with SD and PD were 3.54 and 3.23, respectively (Fig. 2A). Thus, the average miR-522-3p value of patients who did not respond to paclitaxel plus platinum-based chemotherapy (SD + PD) was significantly lower than that of those who responded (CR + PR) (7.26 vs. 3.36, p < 0.05, Fig. 2B). Further, the effect of miR-522-3p expression on ovarian cancer prognoses was examined using a public database. Relative to high expression levels, low miR-522-3p expression levels were significantly correlated with poor progression-free survival (PFS) in the PROGmiRV2 database^11^ (927 days vs. 667 days, Fig. 2C), as well as poor overall survival (OS) in the PROGmiRV2 database^11^ and Kaplan–Meier Plotter database^12,13^ (Fig. 2C,D). These clinical data indicate that lower miR-522-3p expression levels may be associated with the response to paclitaxel plus platinum-based chemotherapy and poor prognoses in ovarian cancer.

### miR-522-3p modulates sensitivity to paclitaxel

**Figure 3:**
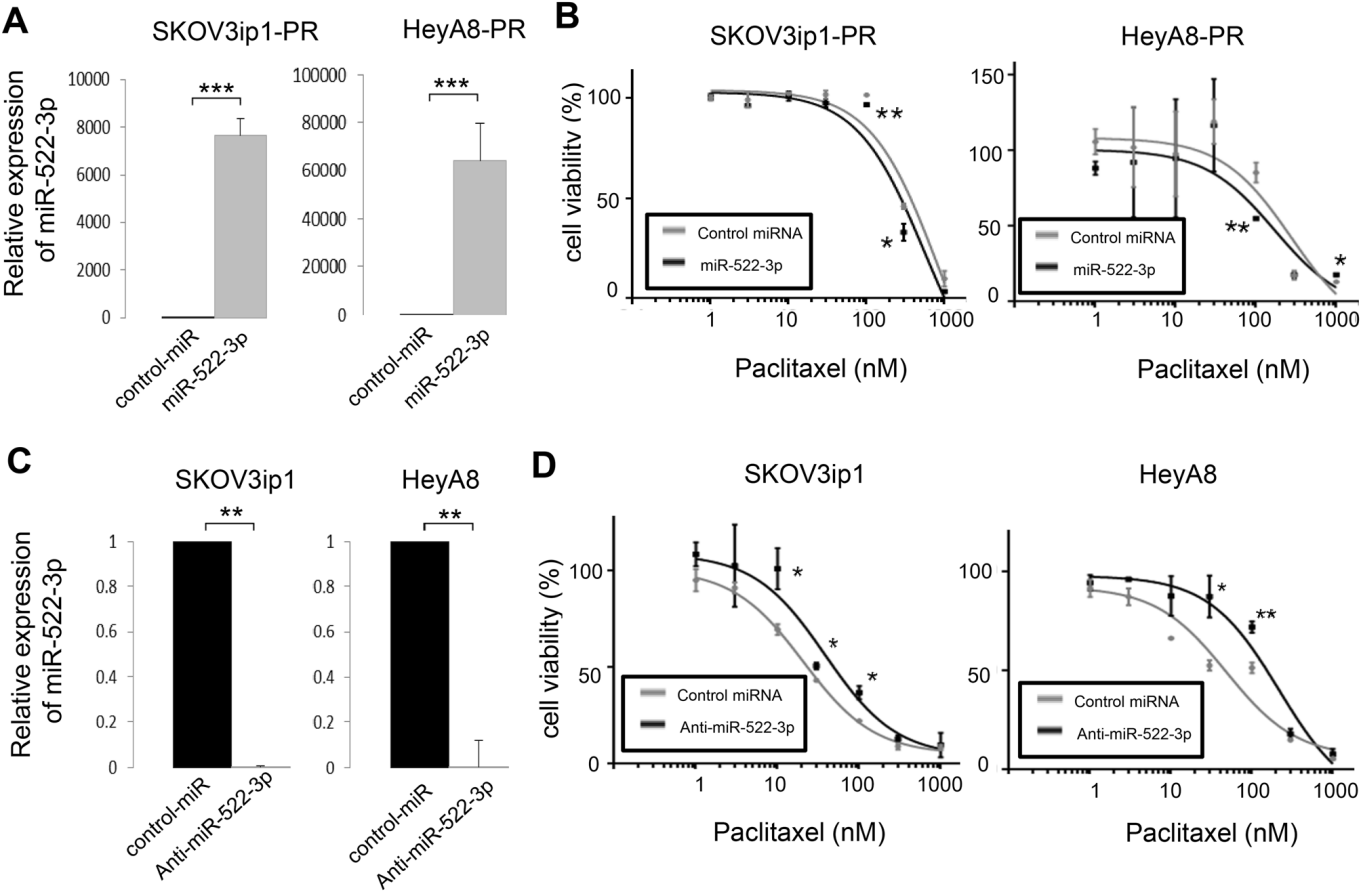
miR-522-3p expression is associated with paclitaxel sensitivity in ovarian cancer cell lines. **(A)** Cells were transfected with pre-miR-522-3p or control-miR, and the expression of miR-522-3p relative to RNU6B was calculated 24 h later using miRNA quantitative reverse transcription polymerase chain reaction (RT-qPCR) and the 2^-ΔΔCT^ method (results shown as relative fold differences). **(B)** At 24 h after transfection with miR-522-3p or control-miR, the cells were treated using paclitaxel for 48 h (HeyA8-PR) or 72 h (SKOV3ip1-PR) and then the cell viability was assessed based on the MTS assay. **(C)** Cells were transfected with anti-miR-522 or control-miR for 24 h before miRNA RT-qPCR was performed. **(D)** At 24 h after transfection with anti-miR-522 or control-miR, the cells were treated using paclitaxel for 48 h (HeyA8) or 72 h (SKOV3ip1), and then cell viability was assessed based on the MTS assay. Experiments were performed in triplicate. Data are presented as mean ± standard deviation from three independent experiments. *P < 0.05, **P < 0.01, ***P < 0.001.

Cell viability was assessed after either the restoration or silencing of miR-522-3p, based on the transfection of SKOV3ip1-PR and HeyA8-PR cells with precursor miR-522-3p or control miRNA. The quantitative miRNA RT-PCR results confirmed successful transfection (SKOV3ip1-PR: 7651-fold, HeyA8-PR: 64020-fold; Fig. 3A), and the MTS assay revealed that the miR-522-3p-transfected paclitaxel-resistant cells were more sensitive to paclitaxel than their corresponding controls. In the SKOV3ip1-PR cells, the IC_50_ values were 334.0 nM for control miRNA and 191.7 nM for miR-522-3p. In the HeyA8-PR cells, the corresponding values were 220.6 nM for control miRNA and 188.1 nM for miR-522-3p (Fig. 3B). However, transfection with anti-miR-522-3p or control miRNA led to the successful inhibition of miR-522-3p expression based on the quantitative RT-PCR (RT-qPCR) results (SKOV3ip1-PR: 0.002-fold, HeyA8-PR: 0.004-fold, Fig. 3C). The parental cells transfected with anti-miR-522-3p also exhibited greater resistance to paclitaxel than the corresponding controls (Fig. 3D). In the SKOV3ip1 cells, the IC_50_ values were 20.8 nM for control miRNA and 38.5 nM for anti-miR-522-3p, while in the HeyA8-PR cells, the corresponding values were 48.3 nM for control miRNA and 216.7 nM for anti-miR-522. These results indicated that miR-522-3p modulated sensitivity to paclitaxel in ovarian cancer cells.

### E2F2 is a direct target for miR-522-3p

**Figure 4:**
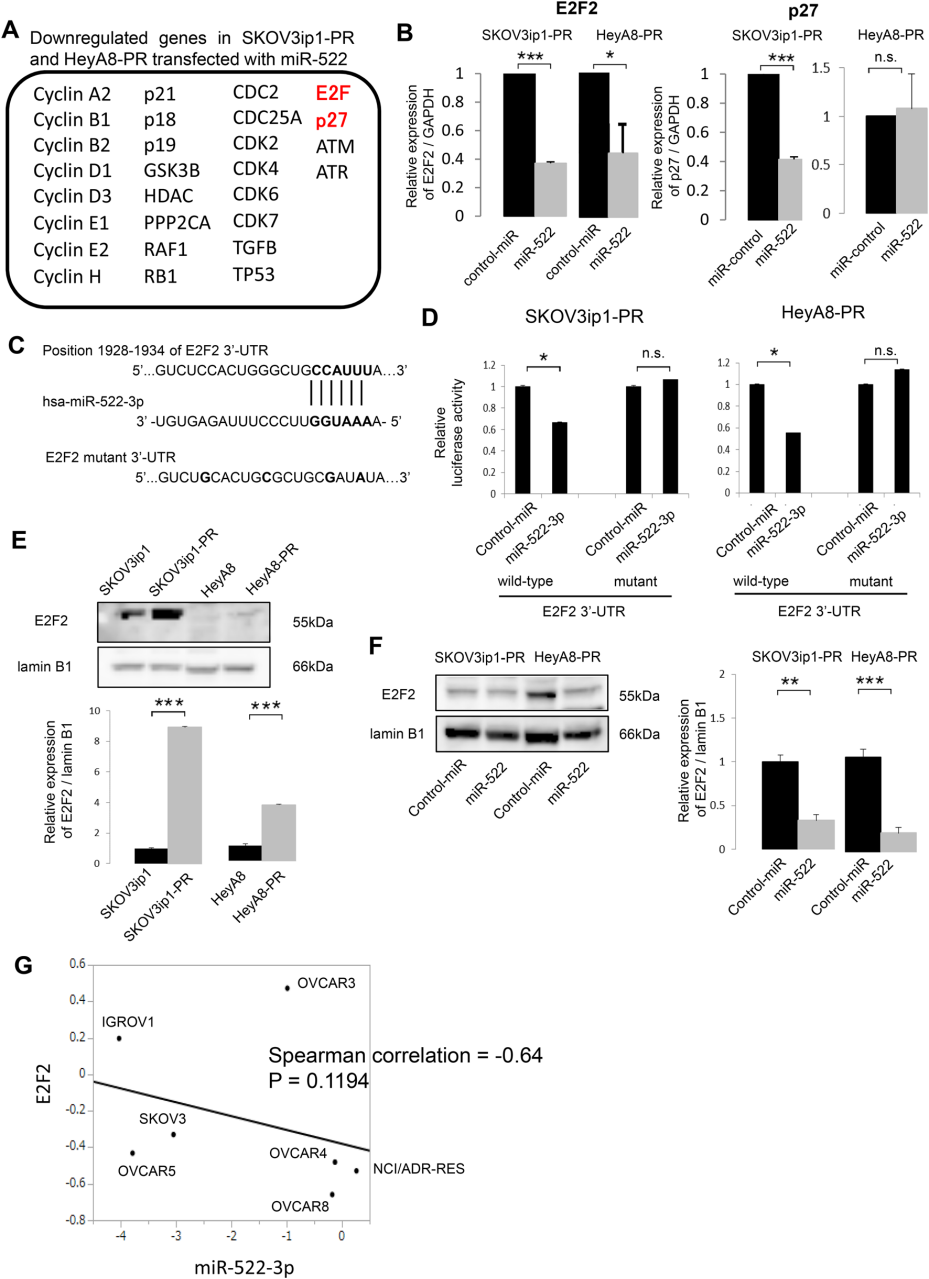
E2F2 is a direct target of miR-522-3p and its expression is upregulated in paclitaxel-resistant cells. **(A)** The list of downregulated genes in the SKOV3ip1-PR and HeyA8-PR cells transfected with miR-522-3p, relative to the cells transfected with control miRNA (red indicates the downregulated genes captured by a biotinylated miR-522-3p mimic based on Ref.^16^). **(B)** The quantitative reverse transcription polymerase chain reaction findings on the relative expression of E2F2 and p27 (vs. control miRNA) in paclitaxel-resistant ovarian cancer cell lines transfected with miR-522-3p. **(C)** Schematic illustration of the predicted E2F2 3′-untranslated region (UTR)-binding site of miR-522-3p. **(D)** Paclitaxel-resistant cells were co-transfected with miRNA precursor (miR-522-3p or control-miR), a luciferase reporter vector containing the wildtype or mutant 3′-UTR of E2F2, and the Renilla luciferase control vector. After 24 h of incubation, the luciferase activity level was measured (normalized to Renilla activity). Data are represented as mean ± standard deviation and were obtained from three independent experiments. **(E)** Western blot results for E2F2 expression in the parental cells and paclitaxel-resistant cells. Lamin B1 was used as a loading control (upper). Densitometric ratio of the expression of E2F2 / lamin B1 (lower). **(F)** Western blot results for E2F2 expression in paclitaxel-resistant cells transfected with miR-522-3p or control-miR (left). Densitometric ratio of the expression of E2F2 / lamin B1 (right). **(G)** Correlation plot extracted from seven ovarian cancer cell lines deposited in the NCI60 microarray data sets. x axis; the average transcript intensity z score of miR-522-3p, y axis; that of miR-E2F2. *P < 0.05, ***P < 0.001, n.s. not significant.

A previous report that focused on the full-length sequence of transcripts captured by a biotinylated miR-522 mimic revealed that miR-522 induced G1 cell arrest by the inhibition of cell cycle-related proteins, such as E2F and p27^14^. Thus, Taqman Gene Expression assays were performed, and we identified 28 cell cycle-related genes in the paclitaxel-resistant cells transfected with miR-522-3p or control-miRNA (Fig. 4A). Validation using RT-PCR revealed that, following the forced expression of miR-522-3p, the E2F2 expression level was significantly downregulated in the SKOV3ip1-PR cells (0.371-fold) and HeyA8-PR cells (0.439-fold). However, although the p27 expression level was significantly downregulated in the SKOV3ip1-PR cells transfected with miR-522-3p (0.414-fold), significant differences were not observed in the HeyA8-PR cells (1.08-fold) (Fig. 4B). Therefore, we focused on E2F2 as a potential target for miR-522-3p, and TargetScan^15^ suggested that there was a putative miR-522-3p target site in the 3′-UTR of the E2F2 mRNA (position 1928–1934 of the E2F2 3′-UTR, Fig. 4C). We constructed the pMIR-REPORT firefly luciferase miRNA expression reporter vector containing this putative miR-522-3p binding site, and the assay revealed significantly lower relative luciferase activity levels in the SKOV3ip1-PR and HeyA8-PR cells transfected with the precursor miR-522-3p (vs. the control miRNA) (Fig. 4D). Site-specific mutation of the target sequence also prevented the downregulation of luciferase activity that was induced by pre-miR-522-3p (Fig. 4D). Furthermore, the E2F2 expression level was significantly higher in the paclitaxel-resistant cells than their parental cells (SKOV3ip1-PR: 8.93-fold, HeyA8-PR: 3.65-fold, Fig. 4E), and the transduction of miR-522-3p decreased the E2F2 expression level in the paclitaxel-resistant cells (SKOV3ip1-PR transfected with miR-522-3p: 0.33-fold, HeyA-8 transfected with miR-522-3p: 0.20-fold, Fig. 4F). These results indicate that E2F2 is a direct target of miR-522-3p, and that its expression was upregulated during the acquisition of paclitaxel resistance. Further, in order to analyze the correlation between miR-522-3p and E2F2 expression in ovarian cancer cells, the US National Cancer Institute’s NCI60 database—which contains a panel of 60 diverse human cancer cell lines, including seven ovarian cancer lines—was used. In the seven registered ovarian cancer cells (IGROV1, OVCAR-3, OVCAR-4, OVCAR-5, OVCAR-8, SKOV-3, and NCI/ADR-RES), the average transcript intensity z scores of miR-522-3p and E2F2 were inversely correlated (Spearman correlation = − 0.64, P = 0.1194, Fig. 4G), suggesting that miR-522-3p is among the key regulators of E2F2 in ovarian cancer cells.

### Downregulation of E2F2 induces paclitaxel sensitivity through the induction of G0/G1 cell cycle arrest

**Figure 5:**
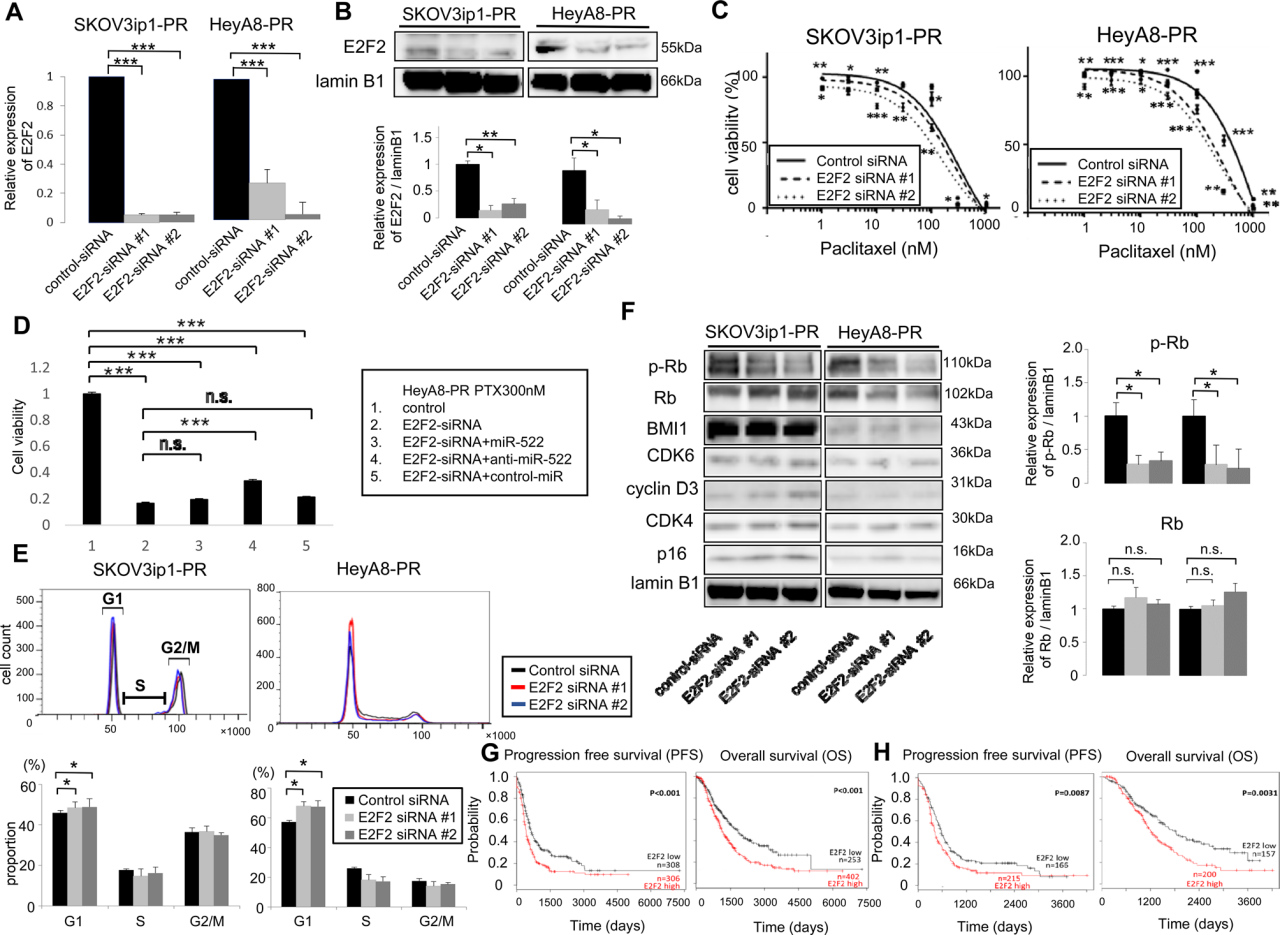
Knockdown of *E2F2* attenuated paclitaxel resistance in paclitaxel-resistant cells thorough G0/G1 arrest. **(A)** The quantitative reverse transcription polymerase chain reaction findings for the relative expression of E2F2 (vs. control siRNA) in paclitaxel-resistant cells transfected with E2F2-siRNAs. **(B)** The western blot findings for E2F2 in paclitaxel-resistant cells transfected with control-siRNA or E2F2-siRNAs (left, SKOV3ip1-PR cells; right, HeyA8-PR cells) (upper). Densitometric ratio of the expression of E2F2 / lamin B1 (lower). **(C)** At 24 h after transfection with control-siRNA or E2F2-siRNAs, the cells were treated using paclitaxel for 48 h (HeyA8-PR) or 72 h (SKOV3ip1-PR) before the cell viability was assessed based on the MTS assay. MTS assays were performed in triplicate. Data are represented as mean ± standard deviation (SD) and were obtained from three independent experiments. **(D)** After transfection with control-miR, E2F2-siRNA, E2F2-siRNA + miR-522, E2F2-siRNA + anti-miR-522 or E2F2-siRNA + control-miR, the HeyA8-PR cells were treated using 300 nM paclitaxel for 48 h and then cell viability was assessed based on the MTS assay. **(E)** Paclitaxel-resistant cells were transfected with control-siRNA or *E2F2*-siRNAs, and stained using propidium iodide. Cell cycle findings were evaluated based on flow cytometry (upper: representative flow histograms, lower: percentages of cells in G1, S, and G2/M). Data are represented as mean ± SD and were obtained from three independent experiments. **(F)** Western blot findings of the expression of E2F2 and cell cycle-related proteins in paclitaxel-resistant cells transfected with control-siRNA or E2F2-siRNAs (left: SKOV3ip1-PR cells, right: HeyA8-PR cells) (left). Densitometric ratio of the expression of phospho-retinoblastoma protein (RB) and RB / lamin B1 (right). **(G)** The Kaplan–Meier curves for progression-free survival (PFS; left) and overall survival (OS; right) according to high or low E2F2 expression among 657 ovarian cancer patients. **(H)** Kaplan–Meier curves for PFS (left) and OS (right) according to high and low E2F2 expression among 381 patients who received chemotherapy that included paclitaxel. Data are presented as mean ± SD from three independent experiments. *P < 0.05, **P < 0.01, ***P < 0.001.

The two siRNAs specific to E2F2 were successfully transfected based on the RT-PCR (Fig. 5A) and western blot results (Fig. 5B). The MTS assay revealed that the paclitaxel-resistant cells transfected with E2F2 siRNA were more sensitive to paclitaxel than their corresponding controls. In the SKOV3ip1-PR cells, the IC_50_ values were 319.5 nM for control siRNA, 294.5 nM for E2F2-siRNA #1, and 188.6 nM for E2F2-siRNA #2. In the HeyA8-PR cells, the IC_50_ values were 469.8 nM for control siRNA, 296.6 nM for E2F2-siRNA #1, and 251.2 nM for E2F2-siRNA #2 (Fig. 5C). In order to further analyze the interaction between miR-522-3p and E2F2, HeyA8-PR cells were transfected with E2F2-siRNA, E2F2-siRNA + miR-522, E2F2-siRNA + anti-miR-522, or E2F2-siRNA + control-miR. Thereafter, treatment with 300 nM of paclitaxel was performed for 48 h, and the cell viability was subsequently assessed (Fig. 5D). The cell viability of the HeyA8-PR cells transfected with E2F2-siRNA was significantly lower than that of the control cells, indicating that E2F2 siRNA sensitized cells to paclitaxel. Since E2F2-siRNA alone exerted the drastic inhibition of E2F2 expression, as shown in Fig. 5A, the addition of miR-522 did not induce further sensitization to paclitaxel. On the contrary, the addition of anti-miR-522 significantly increased the cell viability after paclitaxel exposure, suggesting the E2F2 expression is at least partly regulated by miR-522-3p.

Considering that E2F2 is a transcription factor that plays a crucial role in the cell cycle by the promotion of G0/G1 progression^16^, we performed cell cycle analyses based on flow cytometry. The knockdown of E2F2 in the paclitaxel-resistant cells increased the proportion of G1 phase cells in the SKOV3ip1-PR cells (control-siRNA: 45.8%, E2F2-siRNA #1: 48.4%, E2F2-siRNA #2: 48.8%; P < 0.05) and HeyA8-PR cells (control-siRNA: 56.9%, E2F2-siRNA #1: 67.9%, E2F2-siRNA #2: 67.5%; P < 0.05), as well as decreased the proportions of cells in the S and G2/M phases (Fig. 5E). Western blot analysis revealed that E2F2 siRNA inhibited the phosphorylation of RB after *E2F2* knockdown (SKOV3ip1-PR E2F2-siRNA1 and E2F2*-*siRNA2: 0.28-fold and 0.33-fold, respectively; HeyA8-PR E2F2-siRNA1 and E2F2*-*siRNA2: 0.28-fold and 0.23-fold, respectively), without the alteration of the expression of other cell cycle proteins, such as cyclin D3, CDK4, CDK6, p16, and BMI1 (Fig. 5F).

### Prognostic value of E2F2 expression

In the 655 patients with ovarian cancer in the Kaplan–Meier Plotter database^12,17^, a higher E2F2 expression level was significantly associated with poorer PFS and OS values (vs. lower E2F2 expression) (Fig. 5G). Among 381 patients who had received paclitaxel-containing regimens, higher E2F2 expression levels were still significantly associated with poorer PFS and OS values (Fig. 5H), strongly suggesting that the E2F2 expression in ovarian cancer is related to paclitaxel resistance. These findings indicate that E2F2 is involved in the acquisition of paclitaxel resistance, and that its downregulation leads to paclitaxel sensitivity through the induction of G0/G1 cell cycle arrest.

### Transduction of miR-522-3p inhibits E2F2 expression and induces G0/G1 cell cycle arrest

**Figure 6:**
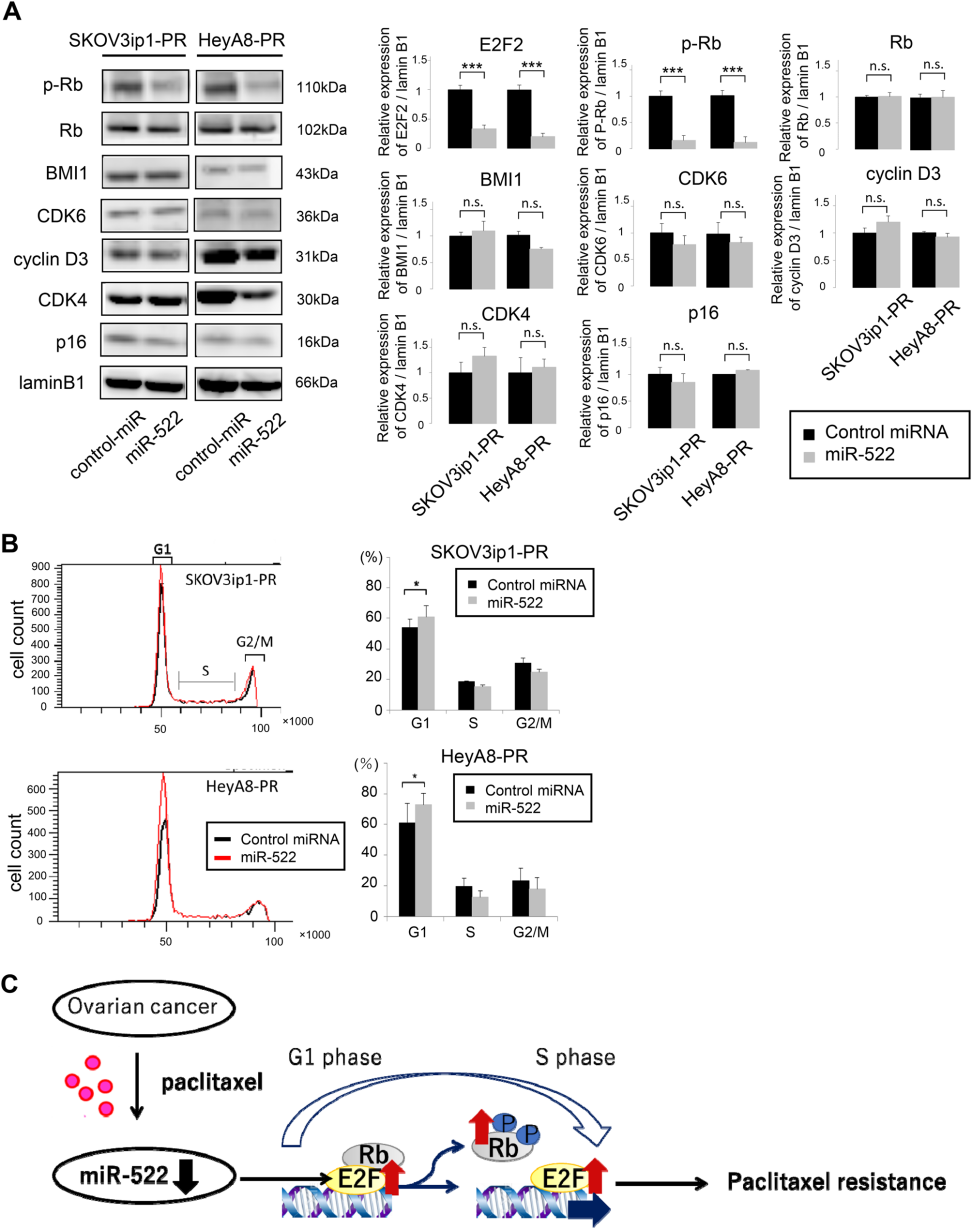
miR-522-3p induces G0/G1 arrest through the downregulation of E2F2. **(A)** Western blot findings for the expression of E2F2 and cell cycle-related proteins in paclitaxel-resistant cells transfected with control-miR or miR-522-3p (left: SKOV3ip1-PR cells, right: HeyA8-PR cells) (left). E2F and lamin blots shown are same as those in Fig. 4F. Densitometric ratio of each expression normalized by lamin B1 expression (right). **(B)** Paclitaxel-resistant cells transfected with control-miR or miR-522-3p and stained using propidium iodide. Cell cycle findings were evaluated based on flow cytometry (left: representative flow histograms, lower: percentage of cells in G1, S, and G2/M phase). Data are represented as mean ± standard deviation and were obtained from three independent experiments. *P < 0.05. **(C)** Schematic diagram showing the involvement of miR-522 downregulation in the acquisition of paclitaxel resistance.

Western blot analyses revealed that the transduction of miR-522-3p into paclitaxel-resistant ovarian cancer cells inhibited the phosphorylation of RB, followed by E2F2 knockdown (SKOV3ip1: 0.16-fold, HeyA8: 0.11-fold), without changes in the expressions of other cell cycle proteins, such as cyclin D3, CDK4, CDK6, p16, and BMI1 (Fig. 6A). The transduction of miR-522-3p also significantly increased the proportion of G1 phase cells in the SKOV3ip1-PR cells (control-miR: 53.9% vs. miR-522-3p: 60.9%; P < 0.05) and HeyA8-PR cells (control-miR: 61.2% vs. miR-522-3p: 72.7%, P < 0.05), as well as decreased the proportions of cells in the S and G2/M phases (Fig. 6B). These results indicate that the restoration of miR-522-3p, the expression of which was downregulated during the acquisition of paclitaxel resistance, downregulated E2F2 expression, and that its downregulation induced G0/G1 arrest, thereby attenuating the degree of paclitaxel resistance.

## Discussion

In this study, we found that the downregulation of miR-522-3p expression was strongly associated with the acquisition of paclitaxel resistance in ovarian cancer cells, while the restoration of miR-522-3p attenuated paclitaxel resistance through the targeting of E2F2. The 2- and 5-year relapse rates associated with advanced ovarian cancer are > 50% and 70%, respectively^1^, highlighting the importance of overcoming chemoresistance. Platinum-paclitaxel chemotherapy is the standard postoperative treatment for ovarian cancer, and the mechanisms behind the acquisition of paclitaxel resistance have been extensively analyzed. However, the mechanisms remain incompletely understood because they are complex and multifactorial, involving the overexpression of drug efflux pumps, mutation in the tubulin gene, tubulin isotype selection, and decreased rates of apoptosis^8^. Various studies have also revealed that miRNA is involved in this process. Li et al. revealed that the dysregulation of miR-27a is involved in the development of paclitaxel resistance in ovarian cancer cells, which involves the regulation of MDR1/P-gp expression through the targeting of HIPK2^18^. Wiemer also reported that the levels of TUBB3 protein are reduced by miR-200c, which increases the sensitivity to microtubule-targeting agents, including paclitaxel^19^. Recently, we showed that the expression of miR-194-5p was downregulated in paclitaxel-resistant ovarian cancer cells, and that the forced expression of miR-194-5p resensitized resistant cells to paclitaxel^10^. Paclitaxel resistance is a major clinical challenge in breast cancer treatment too. Wang et al. revealed that miR-107 expression is downregulated in breast cancer tissues accompanied by the upregulation of tumor protein D-52 (TPD-52). The supplementation of this miRNA enhanced the sensitivity of paclitaxel through the downregulation of TPD-52^20^. Hou et al. reported that low-dose paclitaxel treatment promoted cellular glucose metabolism and intracellular lactate accumulation with upregulated MCT1 (Monocarboxylate transporter 1) expression, contributing to paclitaxel resistance. MCT-1 is a direct target of miR-124, and the transduction of this miRNA led to paclitaxel sensitivity recovery^21^. Therefore, it is obvious that a variety of miRNAs are involved in the acquisition of paclitaxel resistance in several types of cancer, and gaining an understanding of the overall mechanism may aid in overcoming this clinical issue. Platinum resistance is also a critical issue in ovarian cancer treatment, and, moreover, several reports have shown the involvement of miRNAs in this process. Niu et al. demonstrated that the expression of miR-509-3p was significantly downregulated in cisplatin-resistant ovarian cancer tissues, and that this miRNA can sensitize ovarian cancer cells to cisplatin by the downregulation of the expression of Golgi phosphoprotein-3 and wntless Wnt ligand secretion mediator^22^. Dominik et al. revealed that miR-424-3p sensitizes ovarian cancer cells to cisplatin through decreases in the expression of galectin-3^23^. Since miRNAs do not require perfectly complementary target sites, a single miRNA regulates hundreds of genes, and a single oncogene is regulated by hundreds of miRNAs. Therefore, a single miRNA is insufficient for the achievement of clinical goals, and the use of a combination of miRNAs that restore paclitaxel resistance would be ideal for future clinical application. The present study established paclitaxel-resistant ovarian cancer cell lines via continuous exposure to paclitaxel. Using those cell lines, we found that miR-522-3p expression was downregulated during the acquisition of paclitaxel resistance, while miR-522-3p restoration led to the sensitization of paclitaxel-resistant cells to paclitaxel and miR-522-3p inhibition induced paclitaxel resistance in the parental cells. Moreover, we found that *E2F2* was a direct target gene of miR-522-3p and that the transduction of miR-522-3p into paclitaxel-resistant cells induced sensitivity to paclitaxel through the induction of G0/G1 cell cycle arrest by E2F2 downregulation (Fig. 6C).

In this context, miR-522 is a member of the chromosome 19 miRNA cluster (C19MC), which is among the largest miRNA clusters in the human genome, with 46 miRNAs within a 100 kb sequence^24^. C19MC miRNAs have been implicated in various cancers and are considered an oncogenic miRNA cluster^13^. Tan et al. performed full-length sequencing of the transcripts captured by a biotinylated miR-522 mimic, and found that miR-522 induces G1 cell cycle arrest by the inhibition of E2F and TFDP1, as well as the induction of epithelial mesenchymal transition by the upregulation of the expression of related proteins, such as BMI1, FOXA1, and TGIF2^13^. Interestingly, miR-522-3p plays roles as both an oncogenic miRNA and a tumor-suppressor miRNA. Feng et al. demonstrated that miR-522-3p promotes tumorigenesis in human colorectal cancer by targeting the Bloom Syndrome Protein^25^. However, Tianze et al. reported that the downregulation of miR-522 suppressed the proliferation and metastasis of non-small cell lung cancer cells by directly targeting the DENN/MADD domain containing 2D^26^. Zhang et al. also reported that miR-522 contributed to the proliferation of human glioblastoma cells by the suppression of PHLPP1 expression^27^. In contrast, Yang et al. reported that miR-522 reversed the drug resistance of doxorubicin-induced HT29 colon cancer cells by targeting ABCB5^28^. However, we are not aware of any other studies that focused on the role of this miRNA in ovarian cancer. The present study revealed that E2F2 was a direct target of miR-522-3p, and that it played a key role in the acquisition of paclitaxel resistance by the control of the progression from the G1 phase to S phase. In Fig. 4B, the transduction of miR-522-3p downregulated the p27 expression level of the SKOV3ip1-PR cells but not the HeyA8-PR cells. Since it is well known that p27 is regulated by multiple signal transduction pathways in normal and malignant cells such as the FoxO family and the AGC kinase family^29^, the p27 expression level may differ between cells regardless of the regulation by miRNA.

Our data suggest that the restoration of miR-522-3p after paclitaxel exposure may potentially help overcome paclitaxel resistance. Although several miRNA replacement therapies are being evaluated in preclinical trials^30^, the outcomes of a few translational clinical trials have been disappointing so far, based on concerns regarding the optimal delivery system, off-target effects, long-term safety, neurotoxicity, and low bioavailability^31^. The first clinical study that investigated miRNA replacement therapy as cancer treatment was initiated in 2013 for the evaluation of a liposome-formulated miR-34 mimic for metastatic liver cancer treatment^32^; however, that study was terminated because of adverse toxic effects. There are several ongoing clinical trials that are using miRNA mimics with less toxic delivery methods, and the TargomiR trial with miR-16 entered phase II without the induction of toxicity or an adverse immune response^33^.

The present study has several limitations. First, we were unable to provide clinical or pathological evidence that miR-522-3p expression was downregulated after paclitaxel exposure, as recurrent tumor specimens were generally not available after what was predominantly non-surgical treatment. Second, we were unable to examine whether miRNA replacement therapy was effective in vivo, as the paclitaxel-resistant cells could not be inoculated into immunodeficient mice. Thus, further studies are needed to determine whether the use of miR-522-3p as a target can help overcome paclitaxel resistance in clinical settings.

In conclusion, we found that the downregulation of miR-522-3p was strongly associated with the acquisition of paclitaxel resistance in ovarian cancer cells, while its restoration attenuated paclitaxel resistance by targeting E2F2. Thus, miR-522-3p may be a useful therapeutic target for overcoming paclitaxel resistance, although further studies are needed to determine whether this has the potential be an effective and non-toxic miRNA replacement strategy.

## Materials and methods

In this study, all methods were carried out in accordance with relevant guidelines and institutional regulations.

### Materials

Dulbecco’s modified Eagle’s medium (DMEM) was obtained from Nacalai Tesque (Kyoto, Japan). Fetal bovine serum (FBS; #172012), and paclitaxel (T7402) were purchased from Sigma Aldrich (St. Louis, MO). Antibodies targeting *E2F2* (ab70731) were purchased from Abcam (Cambridge, UK). Antibodies targeting RB (sc-50) were obtained from Santa Cruz Biotechnology (Dallas, TX), and those targeting phospho-RB (#9307) were obtained from New England Biolabs (Ipswich, MA). The cell cycle regulation antibody sampler kit (#9932), as well as the antibodies targeting BMI1 (#2830) and CyclinD3 (#2936), were obtained from Cell Signaling (Danvers, MA). Antibodies targeting P16 (#10883-1-AP) and LaminB1 (#66095-1-IG) were obtained from Proteintech Group (Chicago, IL). TRIzol Reagent (#15596-018) was purchased from Life Technologies (Carlsbad, CA).

### Cell culture

The SKOV3ip1 cell line was generously provided by Dr. Ernst Lengyel (University of Chicago, IL), while the HeyA8 cell line was provided by Dr. Anil Sood (MD Anderson Cancer Center, TX). Cells were cultured in DMEM supplemented with 10% FBS and 100 U/mL penicillin/streptomycin, and incubated in 5% CO_2_ with saturated humidity at 37 °C. Cells were authenticated by short tandem repeat DNA profiling performed by Takara-Bio Inc. (Otsu, Japan) and were used within 6 months after resuscitation.

### Establishment of paclitaxel-resistant ovarian cancer cell lines

Paclitaxel-resistant ovarian cancer cell lines were established, as previously described^10^. Two ovarian cancer cell lines (SKOV3ip1 and HeyA8) were exposed to stepwise paclitaxel concentration increases from 1 to 300 nM over a three-month period. The resulting paclitaxel-resistant cell lines, which we named SKOV3ip1-PR and HeyA8-PR, were cultured with paclitaxel-containing culture medium for the maintenance of paclitaxel resistance.

### miRNA RT-qPCR array

Total RNA was collected from the SKOV3ip1, SKOV3ip1-PR, HeyA8, and HeyA8-PR cells using TRIzol, and miRNA expression profiling was performed using the stem loop RT-qPCR-based TaqMan Human MicroRNA Array Set version 2.0 (#4398965; Applied Biosystems, Carlsbad, CA), according to the manufacturer’s protocol. Data were deposited in a public database and assigned the GSE139043 identifier^9^.

### RT-qPCR analysis of miR-522-3p

RT-qPCR was performed using the StepOnePlus Real-Time PCR System (Applied Biosystems, Foster City, CA), as previously described^10^. Briefly, total RNA was extracted using TRIzol and transcribed into cDNA using the TaqMan MicroRNA Reverse Transcription Kit (#4366596; Applied Biosystems). Mature miR-522-3p were assayed using a TaqMan assay (hsa-miR-522-3p; #002413). To normalize the miRNA expression levels, RNU6B (#001093; Applied Biosystems) was used as an endogenous control. Comparative real-time PCR runs were performed in triplicate, and the relative expression levels of miR-522-3p were calculated using the 2^−ΔΔCt^ method.

### Transfection of miRNA

The ovarian cancer cells were transfected with precursor miRNA (pre-hsa-miR-522-3p, #PM12309) or inhibitor miRNA (anti-hsa-miR-522-3p, #AM12309) at a concentration of 30 nM using Lipofectamine 3000 (#L3000-008; Thermo Fisher Scientific) according to the manufacturer’s instructions. Precursor Negative Control #1 (#AM17110) was used as a control. All oligonucleotides were obtained from Thermo Fischer Scientific (Waltham, MA). At 24 h after transfection, the cells were utilized for subsequent procedures.

### Cell viability assay

Ovarian cancer cells (3–5 × 10^3^ cells/well) were seeded onto 96-well plates and cultured for 24 h. The paclitaxel was diluted to a range of concentrations (1–1000 nM) in DMEM supplemented with 2% FBS, and was then added to the wells. The cells were incubated for 48–72 h before 20 μL of MTS solution (3-(4,5-dimethylthiazol-2-yl)-5-(3-carboxymethoxyphenyl)-2-(4-sulfophenyl)-2H-tetrazolium, inner salt) (Promega, Fitchburg, WI) was added to each well. The cells were incubated at 37 °C for 2 h, and the relative cell viability was subsequently assessed by the measurement of the optical density at 490 nm, with viability normalized to the paclitaxel-free control.

### Taqman gene expression assays

Total RNA was collected using TRIzol from the SKOV3ip1-PR and HeyA8-PR cells that were transfected with negative control or pre-hsa-miR-522-3p. Taqman Gene Expression assays were performed using Taqman Array Gene signature 96-well plates (#4391524; Applied Biosystems, Carlsbad, CA) according to the manufacturer’s protocol.

### Western blotting

A total of 3 × 10^5^ cells were plated onto six well plates and lysed using 1 × sample buffer (0.05 M Tris hydrochloride [pH 6.8], 2% sodium dodecyl sulphate [SDS], 10% glycerol, 0.1% bromophenol blue, and 0.1 M dithiothreitol). The cell lysates were separated using 5–20% SDS polyacrylamide gel electrophoresis gels (Wako, Osaka, Japan) and transferred to polyvinylidene difluoride membranes. Next, the membranes were incubated with the following primary antibodies, which were diluted as indicated using tris-buffered saline with 5% bovine serum albumin: anti-laminB1 (1:1000), anti-E2F2 (1:250), anti-P16 (1:500), anti-BMI1 (1:500), anti-Cyclin D3 (1:500), anti-CDK4 (1:500), anti-CDK6 (1:500), anti-RB-control (1:500), and anti-phospho-RB (1:500). The samples were subsequently incubated with the corresponding secondary immunoglobulin G conjugated to horseradish peroxidase. The proteins were then visualized using an electrochemiluminescence system (PerkinElmer Life Science, Waltham, MA), and luminescent images were analyzed using a Luminoimage analyzer (ImageQuant LAS-4000; GE Healthcare Bio-Sciences Corp., Piscataway, NJ).

### Cell cycle analysis

A total of 1 × 10^5^ cells were plated onto six well plates and transfected with negative control or pre-hsa-miR-522-3p. At 24 h after transfection, the cells were collected and fixed overnight in 75% ethanol. Next, the cells were stained for 1 h at 4 °C using 50 µg/mL propidium iodide (P4864, Sigma) in the presence of 100 µg/mL RNase A (Roche). Finally, cell cycle data were collected using a BD FACSCanto II flow cytometer (BD, Franklin Lakes, NJ), and analyzed using BD FACSDiva software (BD).

### Luciferase reporter assay

Luciferase reporter assay was performed as previously described^10^. The *E2F2* 3′-UTR has a region that has been predicted to bind to hsa-miR-522-3p. Synthetic oligonucleotides were created with four copies of the *E2F2* 3′-UTR (GTCTCCACTGGGCTGCCATTTA; bp 1928–1934 of ENST00000361729) or four copies of a mutated version of the sequence (GTCTGCACTGCGCTGCGATATA), as well as with MluΙ and XhoΙ restriction sites at each end. These oligonucleotides were then cloned into the pMIR-REPORT firefly luciferase miRNA expression reporter vector (Applied Biosystems; #AM5795). After 5 × 10^4^ ovarian cancer cells were seeded onto 24-well plates, 0.5 μg of the pMIR-REPORT vector, 0.05 μg of the pRL-TK Renilla luciferase control vector (#E2241; Promega, Madison, WI, USA), and 40 pM of pre-miR-522-3p or the negative control miRNA were co-transfected using Lipofectamine 3000. At 24 h after transfection, luciferase activity was measured using the Dual-Luciferase Reporter Assay System (#E1910; Promega) according to the manufacturer’s instructions. Firefly luciferase activity was normalized to the Renilla luciferase activity.

### Analysis of the prognostic values of miR-522 and E2F2 using a public database

Clinical data on 489 high-grade serous ovarian carcinoma patients deposited in the TCGA database for ovarian cancer (TCGA-OV) were obtained from cBioPortal For Cancer Gneomics (https://www.cbioportal.org/), and the corresponding relative expression of miR-522-3p deposited in the TCGA-OV was obtained from OncomiR Cancer Database (https://www.oncomir.umn.edu/omcd/basic_search.php). Kaplan–Meier curves were used to compare survival outcomes. The prognostic value of miR-522-3p expression was evaluated using the PROGNOSTIC miRNA DATABASE^11,12^ that includes gene expression data and clinical data. The 549 patients with serous ovarian cancer were divided into high-expression and low-expression groups, and the outcomes were compared in terms of OS and PFS. The prognostic value of miR-522-3p expression was also evaluated using the Kaplan–Meier Plotter database^13,14^ that includes gene expression and clinical data on 485 ovarian cancer patients. The prognostic value of *E2F2* expression (probe; 235582_at) was also evaluated using the Kaplan–Meier Plotter^13,18^, based on data from 1,545 ovarian cancer patients, who were divided into high-expression and low-expression groups.

### NCI60 miRNA and mRNA correlation analysis

Normalized Agilent miRNA and mRNA data sets from NCI60 cell lines were obtained from Cellminer (https://discover.nci.nih.gov/cellminer, last accessed March 31, 2020). Spearman’s rank correlation coefficient and the associated P value were calculated between the average transcript intensity z scores of *E2F2* and miR-522-3p.

### Statistical analyses

All statistical analyses were performed using JMP software version 14.0.0 (SAS Institute Japan Ltd., Tokyo, Japan). Unless otherwise stated, the data are presented as mean ± standard deviation (SD), and statistical significance was analyzed using Wilcoxon’s rank sum test. For the RT-qPCR data, the error bars represent SD as calculated by the StepOnePlus Real-Time PCR System. Differences were considered statistically significant at P-values < 0.05. The prognostic values of miR-522 and *E2F2* expression in ovarian cancer patients were analyzed using Kaplan–Meier survival plots, as described above. The results were reported as hazard ratios with their 95% confidence intervals, and log-rank P-values.

## Supplementary information

Supplementary Information 1.

Supplementary Information 2
