# Supplementary material for: Paclitaxel exposure downregulates miR-522 expression and its downregulation induces paclitaxel resistance in ovarian cancer cells
Source: Sci Rep. 2020 Oct 7;10:16755. doi: 10.1038/s41598-020-73785-8 (PMC7542453; doi:10.1038/s41598-020-73785-8)
Supplement: Supplementary file 2 — Supplementary Information 2 [file 41598_2020_73785_MOESM2_ESM.pptx]

## Slide 1
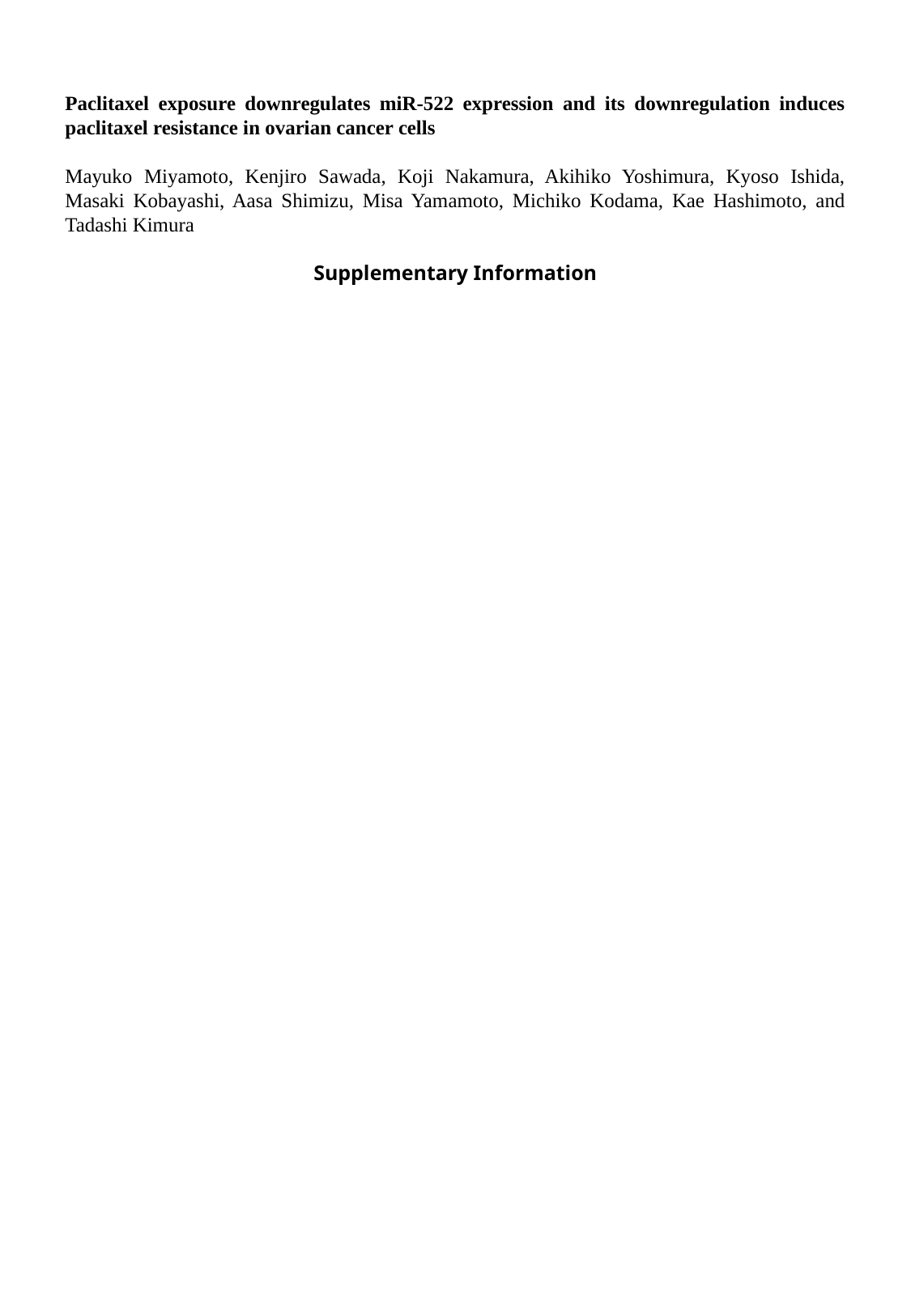

Paclitaxel exposure downregulates miR-522 expression and its downregulation induces paclitaxel resistance in ovarian cancer cells
Mayuko Miyamoto, Kenjiro Sawada, Koji Nakamura, Akihiko Yoshimura, Kyoso Ishida, Masaki Kobayashi, Aasa Shimizu, Misa Yamamoto, Michiko Kodama, Kae Hashimoto, and Tadashi Kimura
Supplementary Information

## Slide 2
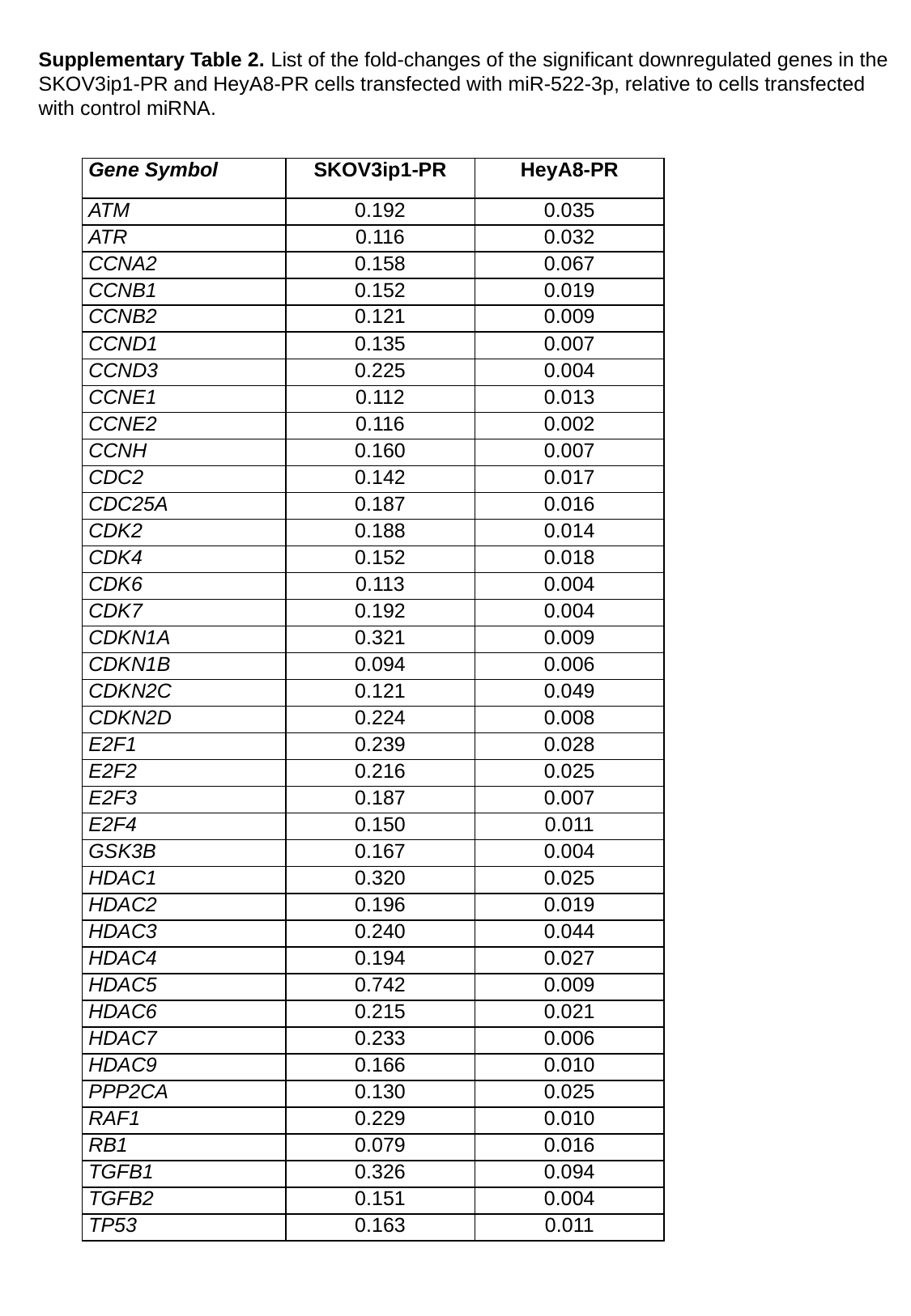

Supplementary Table 2. List of the fold-changes of the significant downregulated genes in the SKOV3ip1-PR and HeyA8-PR cells transfected with miR-522-3p, relative to cells transfected with control miRNA.
| Gene Symbol | SKOV3ip1-PR | HeyA8-PR |
| --- | --- | --- |
| ATM | 0.192 | 0.035 |
| ATR | 0.116 | 0.032 |
| CCNA2 | 0.158 | 0.067 |
| CCNB1 | 0.152 | 0.019 |
| CCNB2 | 0.121 | 0.009 |
| CCND1 | 0.135 | 0.007 |
| CCND3 | 0.225 | 0.004 |
| CCNE1 | 0.112 | 0.013 |
| CCNE2 | 0.116 | 0.002 |
| CCNH | 0.160 | 0.007 |
| CDC2 | 0.142 | 0.017 |
| CDC25A | 0.187 | 0.016 |
| CDK2 | 0.188 | 0.014 |
| CDK4 | 0.152 | 0.018 |
| CDK6 | 0.113 | 0.004 |
| CDK7 | 0.192 | 0.004 |
| CDKN1A | 0.321 | 0.009 |
| CDKN1B | 0.094 | 0.006 |
| CDKN2C | 0.121 | 0.049 |
| CDKN2D | 0.224 | 0.008 |
| E2F1 | 0.239 | 0.028 |
| E2F2 | 0.216 | 0.025 |
| E2F3 | 0.187 | 0.007 |
| E2F4 | 0.150 | 0.011 |
| GSK3B | 0.167 | 0.004 |
| HDAC1 | 0.320 | 0.025 |
| HDAC2 | 0.196 | 0.019 |
| HDAC3 | 0.240 | 0.044 |
| HDAC4 | 0.194 | 0.027 |
| HDAC5 | 0.742 | 0.009 |
| HDAC6 | 0.215 | 0.021 |
| HDAC7 | 0.233 | 0.006 |
| HDAC9 | 0.166 | 0.010 |
| PPP2CA | 0.130 | 0.025 |
| RAF1 | 0.229 | 0.010 |
| RB1 | 0.079 | 0.016 |
| TGFB1 | 0.326 | 0.094 |
| TGFB2 | 0.151 | 0.004 |
| TP53 | 0.163 | 0.011 |

## Slide 3
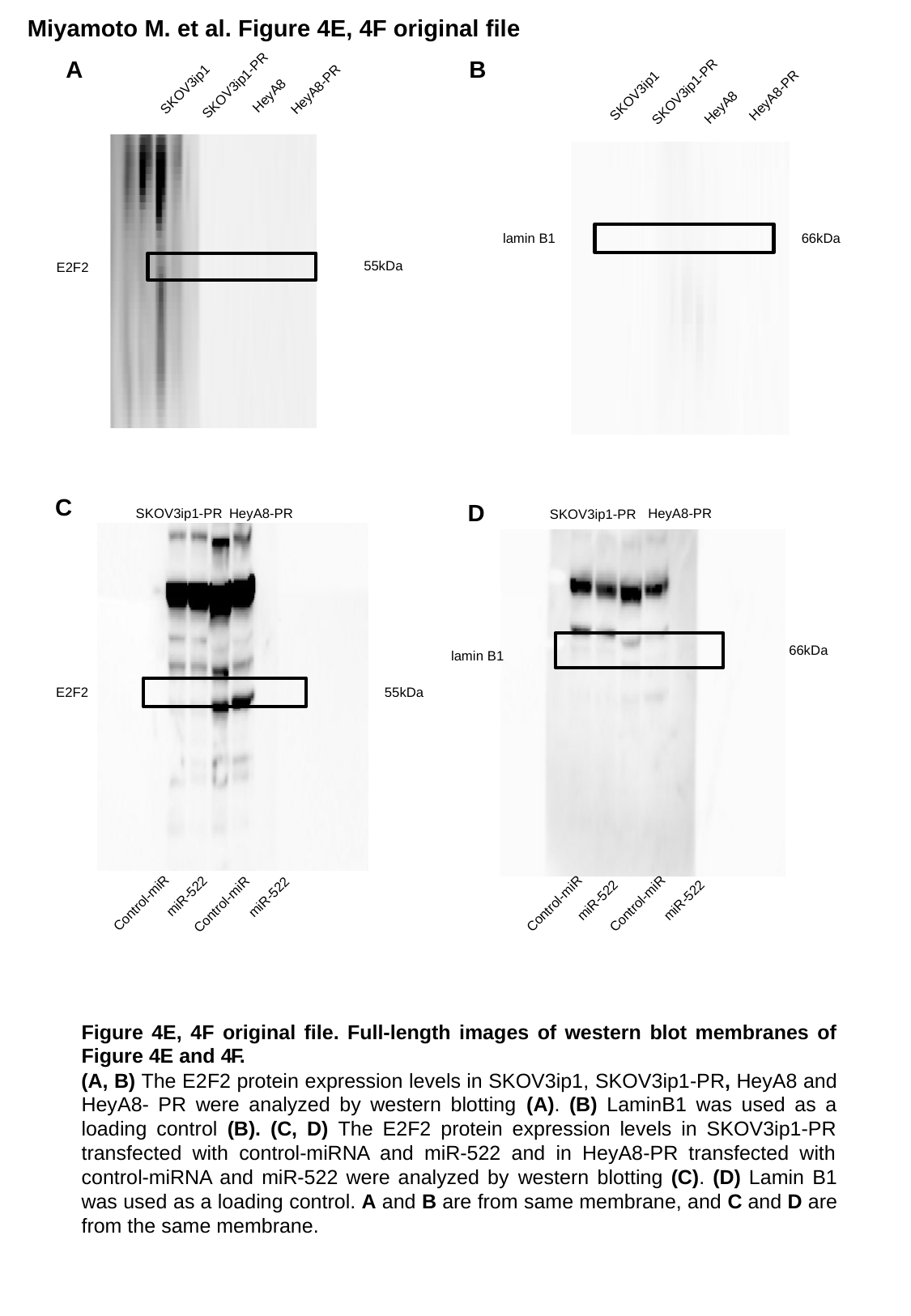

Miyamoto M. et al. Figure 4E, 4F original file
A
B
SKOV3ip1-PR
HeyA8-PR
SKOV3ip1
SKOV3ip1-PR
HeyA8-PR
HeyA8
SKOV3ip1
HeyA8
66kDa
lamin B1
55kDa
E2F2
C
D
HeyA8-PR
HeyA8-PR
SKOV3ip1-PR
SKOV3ip1-PR
66kDa
lamin B1
E2F2
55kDa
miR-522
miR-522
miR-522
miR-522
Control-miR
Control-miR
Control-miR
Control-miR
Figure 4E, 4F original file. Full-length images of western blot membranes of Figure 4E and 4F.
(A, B) The E2F2 protein expression levels in SKOV3ip1, SKOV3ip1-PR, HeyA8 and HeyA8- PR were analyzed by western blotting (A). (B) LaminB1 was used as a loading control (B). (C, D) The E2F2 protein expression levels in SKOV3ip1-PR transfected with control-miRNA and miR-522 and in HeyA8-PR transfected with control-miRNA and miR-522 were analyzed by western blotting (C). (D) Lamin B1 was used as a loading control. A and B are from same membrane, and C and D are from the same membrane.

## Slide 4
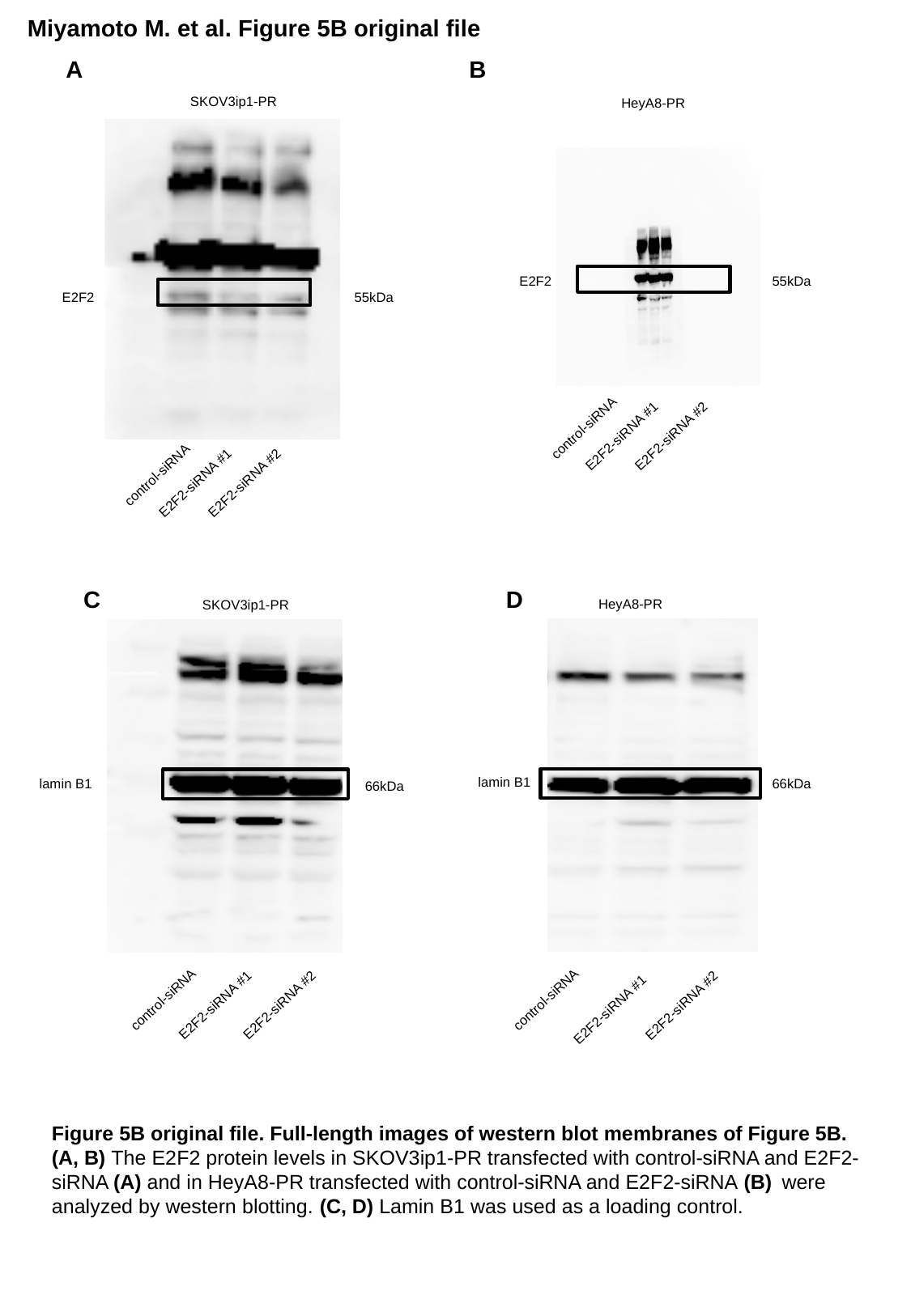

Miyamoto M. et al. Figure 5B original file
A
B
SKOV3ip1-PR
HeyA8-PR
E2F2
55kDa
E2F2
55kDa
control-siRNA
E2F2-siRNA #2
E2F2-siRNA #1
control-siRNA
E2F2-siRNA #2
E2F2-siRNA #1
C
D
HeyA8-PR
SKOV3ip1-PR
lamin B1
lamin B1
66kDa
66kDa
control-siRNA
control-siRNA
E2F2-siRNA #2
E2F2-siRNA #1
E2F2-siRNA #2
E2F2-siRNA #1
Figure 5B original file. Full-length images of western blot membranes of Figure 5B. (A, B) The E2F2 protein levels in SKOV3ip1-PR transfected with control-siRNA and E2F2- siRNA (A) and in HeyA8-PR transfected with control-siRNA and E2F2-siRNA (B) were analyzed by western blotting. (C, D) Lamin B1 was used as a loading control.

## Slide 5
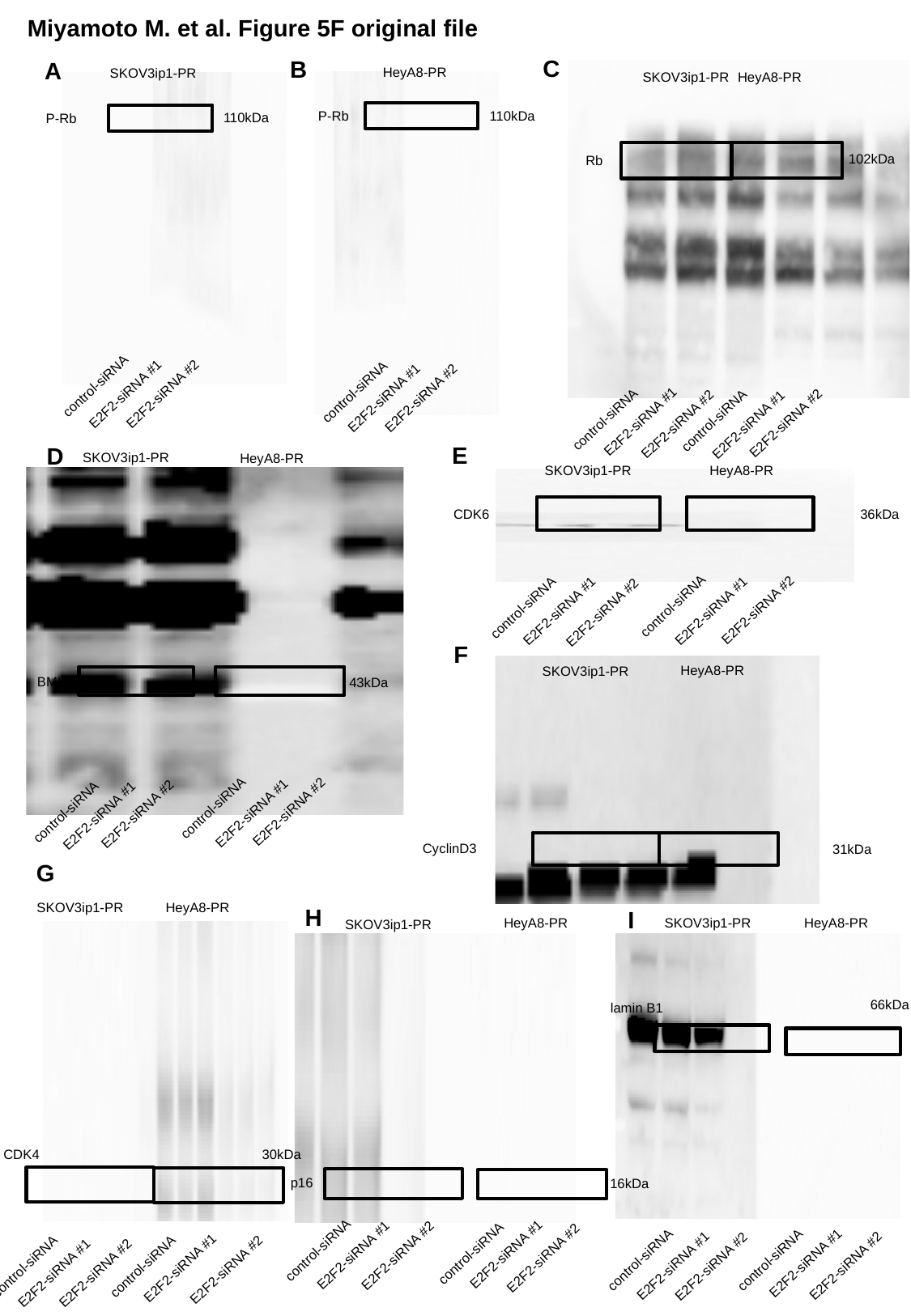

Miyamoto M. et al. Figure 5F original file
C
B
A
HeyA8-PR
SKOV3ip1-PR
SKOV3ip1-PR
HeyA8-PR
P-Rb
110kDa
110kDa
P-Rb
102kDa
Rb
control-siRNA
control-siRNA
E2F2-siRNA #2
E2F2-siRNA #1
E2F2-siRNA #1
E2F2-siRNA #2
control-siRNA
control-siRNA
E2F2-siRNA #1
E2F2-siRNA #2
E2F2-siRNA #2
E2F2-siRNA #1
E
D
SKOV3ip1-PR
HeyA8-PR
SKOV3ip1-PR
HeyA8-PR
CDK6
36kDa
control-siRNA
control-siRNA
E2F2-siRNA #2
E2F2-siRNA #1
E2F2-siRNA #1
E2F2-siRNA #2
F
HeyA8-PR
SKOV3ip1-PR
BMI1
43kDa
control-siRNA
E2F2-siRNA #2
control-siRNA
E2F2-siRNA #1
E2F2-siRNA #2
E2F2-siRNA #1
CyclinD3
31kDa
G
SKOV3ip1-PR
HeyA8-PR
H
I
SKOV3ip1-PR
HeyA8-PR
HeyA8-PR
SKOV3ip1-PR
66kDa
lamin B1
CDK4
30kDa
p16
16kDa
control-siRNA
control-siRNA
E2F2-siRNA #1
E2F2-siRNA #1
E2F2-siRNA #2
E2F2-siRNA #2
control-siRNA
control-siRNA
E2F2-siRNA #1
E2F2-siRNA #2
E2F2-siRNA #1
control-siRNA
control-siRNA
E2F2-siRNA #2
E2F2-siRNA #1
E2F2-siRNA #2
E2F2-siRNA #1
E2F2-siRNA #2

## Slide 6
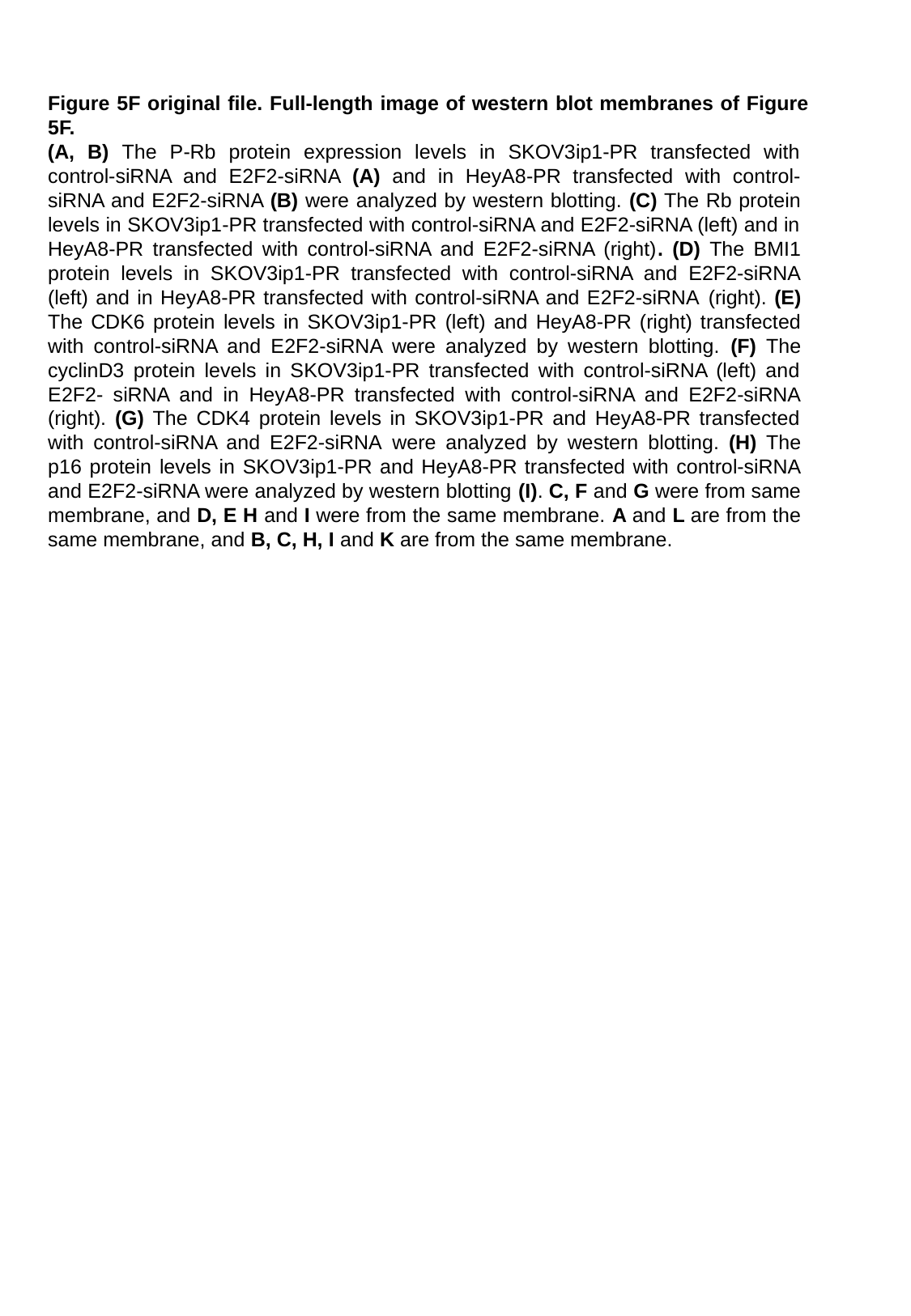

Figure 5F original file. Full-length image of western blot membranes of Figure 5F.
(A, B) The P-Rb protein expression levels in SKOV3ip1-PR transfected with control-siRNA and E2F2-siRNA (A) and in HeyA8-PR transfected with control-siRNA and E2F2-siRNA (B) were analyzed by western blotting. (C) The Rb protein levels in SKOV3ip1-PR transfected with control-siRNA and E2F2-siRNA (left) and in HeyA8-PR transfected with control-siRNA and E2F2-siRNA (right). (D) The BMI1 protein levels in SKOV3ip1-PR transfected with control-siRNA and E2F2-siRNA (left) and in HeyA8-PR transfected with control-siRNA and E2F2-siRNA (right). (E) The CDK6 protein levels in SKOV3ip1-PR (left) and HeyA8-PR (right) transfected with control-siRNA and E2F2-siRNA were analyzed by western blotting. (F) The cyclinD3 protein levels in SKOV3ip1-PR transfected with control-siRNA (left) and E2F2- siRNA and in HeyA8-PR transfected with control-siRNA and E2F2-siRNA (right). (G) The CDK4 protein levels in SKOV3ip1-PR and HeyA8-PR transfected with control-siRNA and E2F2-siRNA were analyzed by western blotting. (H) The p16 protein levels in SKOV3ip1-PR and HeyA8-PR transfected with control-siRNA and E2F2-siRNA were analyzed by western blotting (I). C, F and G were from same membrane, and D, E H and I were from the same membrane. A and L are from the same membrane, and B, C, H, I and K are from the same membrane.

## Slide 7
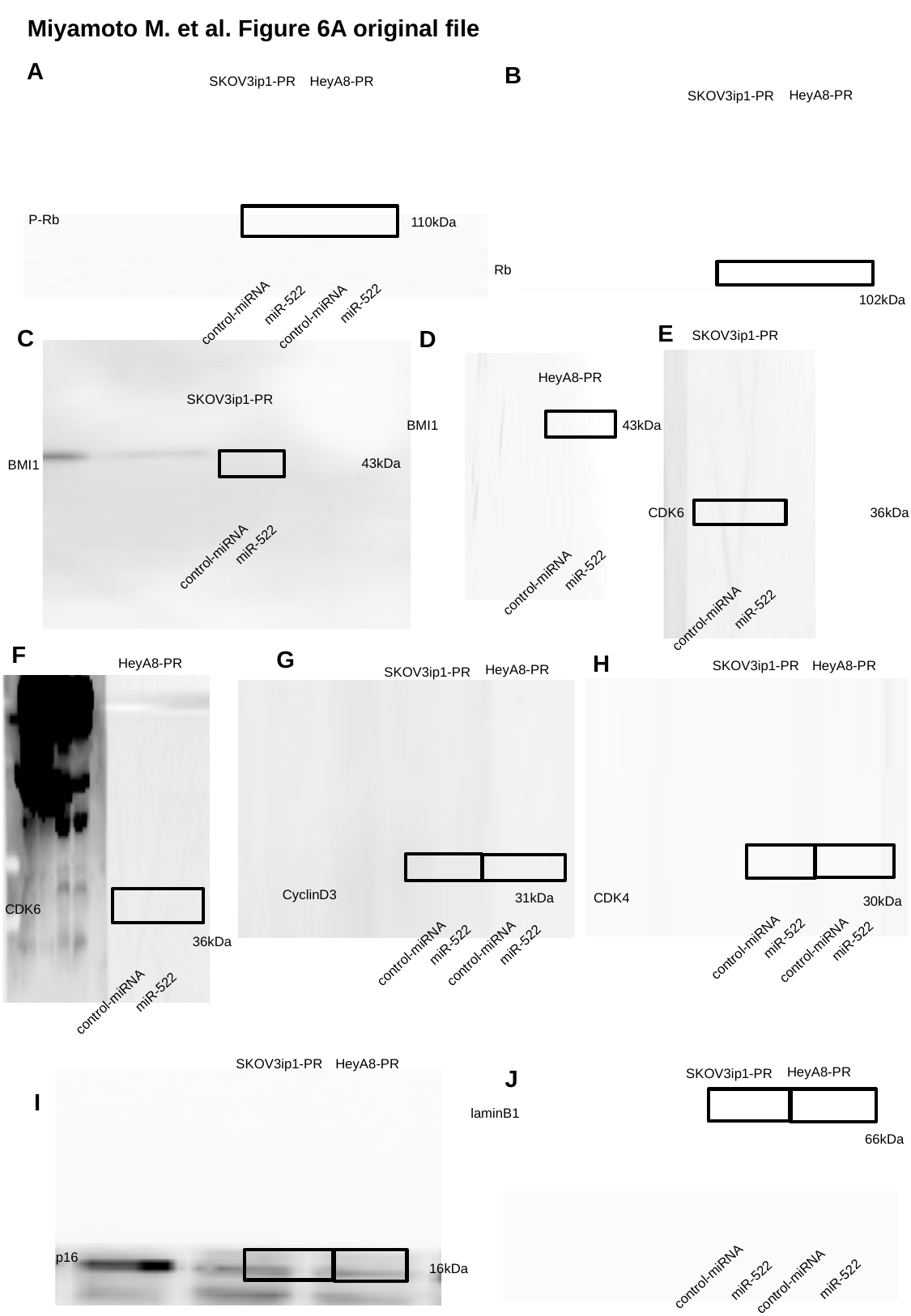

Miyamoto M. et al. Figure 6A original file
A
B
HeyA8-PR
SKOV3ip1-PR
HeyA8-PR
SKOV3ip1-PR
P-Rb
110kDa
Rb
102kDa
miR-522
miR-522
control-miRNA
control-miRNA
E
C
D
SKOV3ip1-PR
HeyA8-PR
SKOV3ip1-PR
BMI1
43kDa
43kDa
BMI1
CDK6
36kDa
miR-522
control-miRNA
miR-522
control-miRNA
miR-522
control-miRNA
F
G
H
HeyA8-PR
HeyA8-PR
SKOV3ip1-PR
HeyA8-PR
SKOV3ip1-PR
CyclinD3
31kDa
CDK4
30kDa
CDK6
miR-522
miR-522
36kDa
miR-522
miR-522
control-miRNA
control-miRNA
control-miRNA
control-miRNA
miR-522
control-miRNA
SKOV3ip1-PR
HeyA8-PR
HeyA8-PR
J
SKOV3ip1-PR
I
laminB1
66kDa
p16
16kDa
control-miRNA
miR-522
miR-522
control-miRNA

## Slide 8
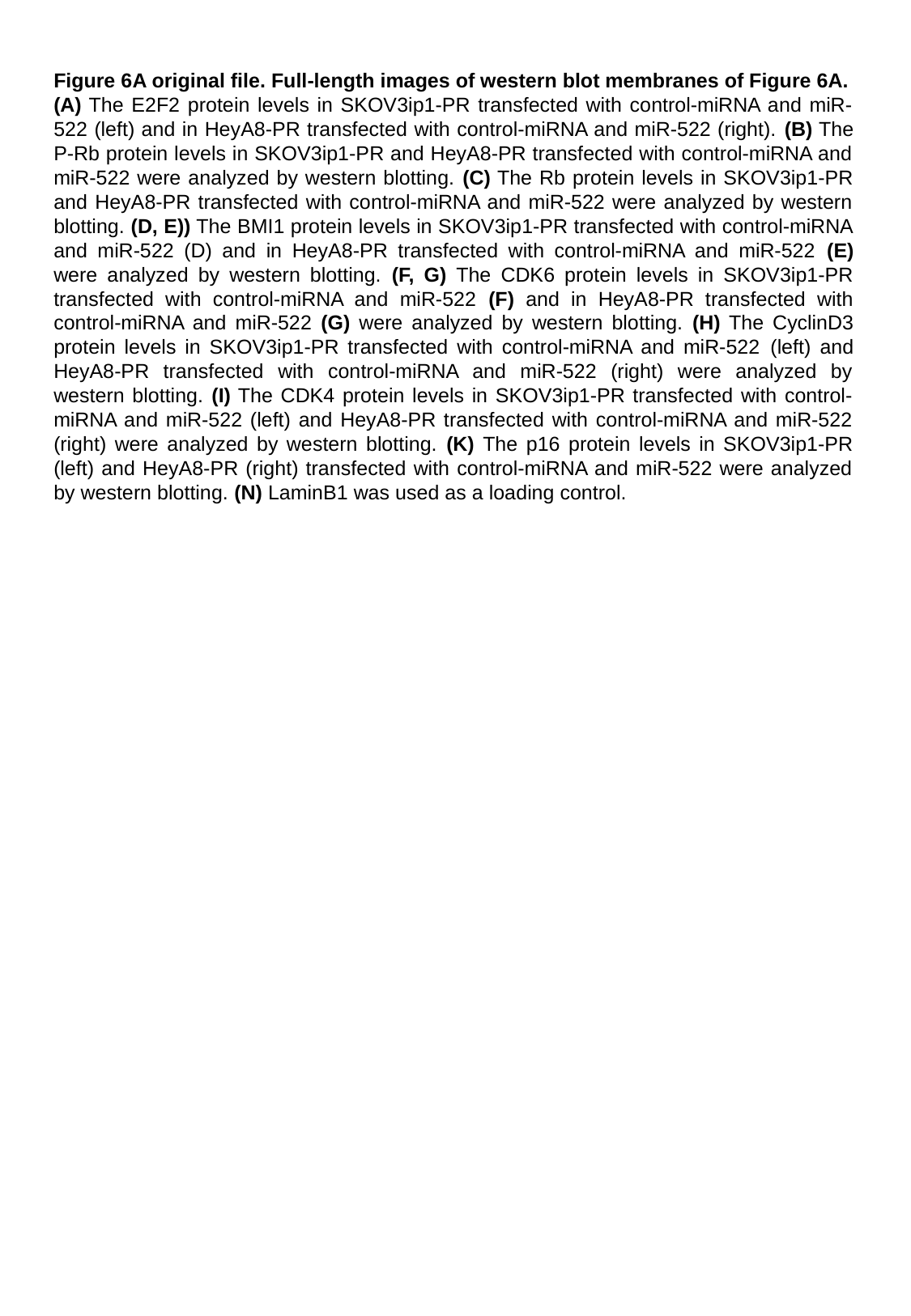

Figure 6A original file. Full-length images of western blot membranes of Figure 6A.
(A) The E2F2 protein levels in SKOV3ip1-PR transfected with control-miRNA and miR-522 (left) and in HeyA8-PR transfected with control-miRNA and miR-522 (right). (B) The P-Rb protein levels in SKOV3ip1-PR and HeyA8-PR transfected with control-miRNA and miR-522 were analyzed by western blotting. (C) The Rb protein levels in SKOV3ip1-PR and HeyA8-PR transfected with control-miRNA and miR-522 were analyzed by western blotting. (D, E)) The BMI1 protein levels in SKOV3ip1-PR transfected with control-miRNA and miR-522 (D) and in HeyA8-PR transfected with control-miRNA and miR-522 (E) were analyzed by western blotting. (F, G) The CDK6 protein levels in SKOV3ip1-PR transfected with control-miRNA and miR-522 (F) and in HeyA8-PR transfected with control-miRNA and miR-522 (G) were analyzed by western blotting. (H) The CyclinD3 protein levels in SKOV3ip1-PR transfected with control-miRNA and miR-522 (left) and HeyA8-PR transfected with control-miRNA and miR-522 (right) were analyzed by western blotting. (I) The CDK4 protein levels in SKOV3ip1-PR transfected with control-miRNA and miR-522 (left) and HeyA8-PR transfected with control-miRNA and miR-522 (right) were analyzed by western blotting. (K) The p16 protein levels in SKOV3ip1-PR (left) and HeyA8-PR (right) transfected with control-miRNA and miR-522 were analyzed by western blotting. (N) LaminB1 was used as a loading control.
